# Caveolin1 protects against diet induced hepatic lipid accumulation in mice

**DOI:** 10.1371/journal.pone.0178748

**Published:** 2017-06-01

**Authors:** Meng Li, Dahua Chen, Haixiu Huang, Jiewei Wang, Xingyong Wan, Chengfu Xu, Chunxiao Li, Han Ma, Chaohui Yu, Youming Li

**Affiliations:** Department of Gastroenterology, the First Affiliated Hospital, College of Medicine, Zhejiang University, Hangzhou, China; East Tennessee State University, UNITED STATES

## Abstract

**Background and aim:**

Caveolin1 (CAV1) is involved in lipid homeostasis and endocytosis, but little is known about the significance of CAV1 in the pathogenesis and development of nonalcoholic fatty liver disease (NAFLD). This study aimed to determine the role of CAV1 in NAFLD.

**Methods:**

Expression of CAV1 in the *in vitro* and *in vivo* models of NAFLD was analyzed. The effects of CAV1 knockdown or overexpression on free fatty acid (FFA)-induced lipid accumulation in L02 cells and AML12 cells were determined. CAV1 knockout (CAV1-KO) mice and their wild-type (WT) littermates were subjected to a high fat diet (HFD) for 4 weeks, and the functional consequences of losing the CAV1 gene and its subsequent molecular mechanisms were also examined.

**Results:**

Noticeably, CAV1 expression was markedly reduced in NAFLD. CAV1 knockdown led to the aggravation of steatosis that was induced by FFA in both L02 cells and AML12 cells, while CAV1 overexpression markedly attenuated lipid accumulation in the cells. Consistent with CAV1 repression in the livers of HFD-induced mice, the CAV1-KO mice exhibited more severe hepatic steatosis upon HFD intake. In addition, increased cholesterol levels and elevated transaminases were detected in the plasma of CAV1-KO mice. The protein expression of SREBP1, a key gene involved in lipogenesis, was augmented following CAV1 suppression in FFA-treated hepatocytes and in the livers of HFD-fed CAV1-KO mice.

**Conclusions:**

CAV1 serves as an important protective factor in the development of NAFLD by modulating lipid metabolism gene expression.

## Introduction

Nonalcoholic fatty liver disease (NAFLD), which encompasses a spectrum from simple hepatic steatosis to steatosis combined with varying degrees of inflammation and fibrosis, has become one of the most common liver diseases around the world [1]. The initial hepatic lipid accumulation is regarded as the “first hit” (the ‘two-hit’ theory) [2], owing to an imbalance of normal hepatic lipid metabolism, which is characterized as excessive lipid influx, decreased lipid clearance, or both [3]. However, the molecular mechanisms underlying hepatic fat accumulation and the trigger for the subsequent hepatocyte injury remain unclear. To better understand the potential pathogenesis and mechanisms of the disease is imperative for developing novel treatment strategies for NAFLD.

Caveolin-1 (CAV1) is the 21–24 kDa major and essential structural protein of caveolae, which remain one of the most intriguing and enigmatic organelles in the cell [4]. Caveolae, 50–100 nm flask/tube-shaped invaginations of the plasma membrane, are specialized membrane microdomains that are formed as a result of the localized accumulation of cholesterol, glycosphingolipids, and CAV1 [5]. Previous research has shown that caveolae and CAV1 participate in cellular transport processes, including endocytosis, membrane traffic, and cholesterol efflux [6]. In addition, CAV1 has been implicated in signal transduction, the control of glucose homeostasis and lipid metabolism regulation [7,8]. Nevertheless, there is controversy concerning the role of CAV1 in lipogenesis and obesity because CAV1 gene expression was found to be significantly decreased in the visceral adipose tissue of obese subjects [9] while the findings of another study were contradictory[10]. One study indicated that CAV1-knockout (CAV1-KO) mice were resistant to high fat diet (HFD)-induced obesity [4]. NAFLD is frequently associated with obesity, dyslipidemia, type 2 diabetes mellitus (T2DM) and insulin resistance (IR), a group of disorders that constitute the metabolic syndrome [11]. The liver plays a central role in whole-body glucose and lipid homeostasis, but studies regarding the role of CAV1 in this organ are still limited. It has been proposed that CAV1 is important in the modulation of lipid metabolism during liver regeneration in mice [12]. In addition, the lack of CAV1 alters hepatocyte energy metabolism homeostasis under physiological and pathological conditions [13]. Despite this recent progress, the precise role of CAV1 in the development of NAFLD remains unclear.

Considering the functional aspects of CAV1, including its active site for downstream signaling molecules and its roles in lipid transport and nutrient storage [14], we hypothesized that CAV1 plays an important role in the pathogenesis and development of NAFLD potentially by modulating hepatic lipid metabolism. To investigate these aims, we used different techniques including siRNA transient transfection and the CRISPR-Cas9 system combined with adenovirus recombination as well as lentiviruses to achieve CAV1 gene knockdown or overexpression in human and mouse hepatocyte cell lines cultured with free fatty acids (FFAs); we also used HFD-fed CAV1-KO mice. We sought to obtain novel insights into the role of CAV1 in pathological hepatic steatosis. Additionally, we investigated the molecular mechanisms by which CAV1 performs its roles during these processes.

## Materials and methods

### Reagents

Dulbecco’s modified Eagle’s medium (DMEM), fetal bovine serum (FBS), and antibiotics were obtained from Gibco (Grand Island, NY). Dexamethasone, insulin, transferrin and selenium (ITS) were purchased from Sigma (Taufkirchen, Germany). The antibodies used in this study were as follows: anti-Caveolin-1 antibody (ab2910), anti-SREBP1 antibody (ab3259), and anti-fatty acid synthase antibody (ab128856) were obtained from Abcam (Cambridge, UK); rabbit mAb acetyl-CoA carboxylase (C83B10) was from Cell Signaling Technology (Beverly, MA, #3676); the mouse monoclonal GAPDH antibody was from Proteintech, (Chicago, IL60004-1-1g); and goat anti-rabbit IgG-HRP (sc-2004) and goat anti-mouse IgG-HRP (sc-2005) were obtained from Santa Cruz Biotechnology (Santa Cruz, CA).

### Animals

All animals used in this study, including the C57BL/6J mice and CAV1-KO mice, were maintained at 23 ± 2°C in a 12-h light/12-h dark cycle at the Experimental Animal Center in Zhejiang Province (Hangzhou, China). CAV1-KO and their corresponding wild-type (WT) littermates, on the same genetic background strain CAV-1^tm1Mls^, were obtained from Jackson Laboratories (Bar Harbor, ME, USA). The mice were given free access to water and a standard rodent diet prior to the study. In the first part of the experiment, specific pathogen-free C57BL/6 mice were randomly divided into two groups that were fed standard chow diet (SCD) which was provided from the Medical Science Institution of Zhejiang Province (Hangzhou, China), or HFD (D12492; Research Diets, New Brunswick, NJ) for 8 and 12 weeks. In the second part of the experiment, ten-week-old male WT or CAV1-KO mice fed on SCD were used to evaluate the potential differences in metabolic parameters between two groups. In the third part of the experiment, eight-week-old male WT or CAV1-KO mice were fed an HFD for 4 weeks. All animal experiments were performed according to the guidelines approved by the Animal Care and Use Committee of the First Affiliated Hospital College of Medicine at Zhejiang University (Permit Number: 2016–370).

### Cell culture

The human normal hepatocyte L02 cell line and the mouse hepatocyte AML12 cell line were used in this study. L02 hepatocytes were obtained from the Chinese Academy of Science (GNHu 6; Shanghai, China) and cultured in DMEM containing 10% FBS and 1% antibiotic (100 U/ml streptomycin and 100 U/ml penicillin). AML12 cells were obtained from the American Type Culture Collection and were grown in DMEM/Ham’s F12 media supplemented with 10% FBS mixed with 40-ng/mL dexamethasone and ITS. To establish a cellular model of hepatic steatosis, cells were treated for 48 h with a mixture of FFA that included oleate and palmitate in a final ratio of 2:1 and at a final concentration of 1 mM, respectively.

### CAV1 silencing in L02 cells

For CAV1 silencing *in vitro*, L02 cells were transfected with a human CAV1-specific small interference RNA (siCAV1) or scrambled siRNA as a negative control (NC) (both purchased from GenePharma, Shanghai, China) using lipofectamine 2000 (Invitrogen, Shanghai, China) following the manufacturer’s protocol. After a 24-h transfection, cells were exposed to FFA for an additional 48 h. The CAV1 siRNA oligonucleotides were as follows:

Sense, 5’-AUUUCUUUCUGCAAGUUGAUGCGGA-3’ and

Anti-sense, 5’-UCCGCAUCAACUUGCAGAAAGAAAU-3’. The scrambled siRNA oligonucleotides were as follows:

Sense, 5’- UUCUUCGAACGUGUCACGUTT-3’

Anti-sense, 5’- ACGUGACACGUUCGGAGAATT-3’.

### CAV1 knockdown mediated by the adenovirus-based CRISPR /Cas9 system in AML12 cells

To stably knock down CAV1 expression in the mouse hepatocyte AML12 cell line, we developed an adenovirus-based CRISPR (clustered regularly interspaced short palindromic repeat)/Cas9 system for gene editing through standard methods that were previously reported [15]. With two single-guide RNAs (sgRNAs) targeting to a specific site, the mutant Cas9 can generate a double-strand break at the targeted site. Genomic DNA was extracted from cells or liver tissues with an established protocol.

### CAV1 overexpression *in vitro*

For CAV1 overexpression *in vitro*, L02 cells were infected with adenoviruses expressing CAV1 protein (Ad-CAV1) (Invitrogen, Shanghai, China). L02 cells treated with adenovirus expressing green fluorescent protein (GFP) served as negative controls (Ad-GFP) (Invitrogen, Shanghai, China). After transfection, L02 cells were treated with FFA for 48 h in the presence of Ad-GFP or Ad-CAV1. Stable CAV1-overexpressing AML12 cell lines were developed using lentiviral particles containing full-length mouse caveolin-1 (Lenti-CAV1). Lenti-GFP served as a negative control. Puromycin was added to eliminate non-transduced cells.

### Histological analysis

Livers were fixed in 4% neutral buffered formalin and embedded in paraffin. Then, liver sections were cut and stained with hematoxylin and eosin (H&E). For the detection of neutral lipids, liver cryosections were stained with Oil Red O according to standard procedures. Cells grown on glass cover slips in 6-well plates were washed with PBS and fixed with 10% neutral formalin followed by staining with Oil Red O and hematoxylin. Sections were imaged at ×400 magnification (Olympus, Japan). The average integrated optical density (IOD) of lipid droplets stained with Oil Red O from FFA treated cells was measured with an Image-Pro Plus software. Each experiment was performed three times with duplicate wells in each group.

### BODIPY (493/503) staining

Cells grown in 96-well plates were fixed with 4% formaldehyde in PBS and then stained with BODIPY 493/503 (D3922; Invitrogen, Shanghai, China) to visualize lipid droplets for 20 min at room temperature in the dark. Hoechst 33342 (62249; Thermo Fisher Scientific, USA) was used for the fluorescence staining of nuclei. Images were acquired with an automated microscopy platform (Operetta High Content Imaging System; PerkinElmer, Waltham, MA, USA) and analyzed automatically for number of and area covered by lipid droplets to calculate the lipid spot area per cell, expressed as area per pixel (px2) within the cytosol using Harmony 4.1 (PerkinElmer, Waltham, MA, USA). The data shown were from one representative experiment of six independent repeats.

### Biochemical measurements

Intrahepatic and intracellular triglyceride (TG) and total cholesterol (TC) levels were quantified using commercial kits (E1013, E1015; Applygen Technologies Inc., Beijing, China) according to the manufacturer’s instructions. Briefly, collected cells or liver tissue homogenates were treated with lysis buffer on ice. Lysates were heated at 70°C for 10 min (this step was optional for TC test), and centrifuged at 2000 rpm for 5 min at room temperature. The supernatant was then assessed with according working solution. TG and TC values were normalized with the total protein levels. The protein concentration in the resulting lysates was determined using the bicinchoninic acid protein assay kit (Applygen Technologies Inc.). Plasma biochemical parameters including TG, TC, HDL-c, LDL-c, ALT and AST levels were detected using a Hitachi 7600 autoanalyzer (Hitachi, Tokyo, Japan) according to the standard procedures set depending on programmed technical parameters.

### RNA isolation and qRT-PCR

Total RNA was prepared from cell lines or tissues using TRIzol reagent (Invitrogen, Shanghai, China) according to the manufacturer’s instructions. Reverse transcription reactions were performed using the PrimeScript RT reagent Kit (Takara, Tokyo, Japan) for mRNA detection. The resulting cDNA was quantified with the ABI 7500 FAST real-time PCR System (Applied Biosystems, Carlsbad, USA) using SYBR Green (Takara, Japan). Levels of relative expression were calculated and quantified with the 2-ΔΔCt method after normalization with the expression level of GAPDH. The primer sequences of CAV1 and GAPDH were as follows: CAV1 (Mouse) Forward Primer: 5’-CTGAACTTTTCTTCCCACCGC, Reverse Primer: 5’-CTTCAAAGTCAATCTTGACCACGTC-3’; CAV1 (Human) Forward Primer: 5’-GAGGGACATCTCTACACCGTTC-3’, Reverse Primer: 5’-ACTGAATCTCAATCAGGAAGCTCT-3’; GAPDH (Mouse) Forward Primer: 5’-AGGTCGGTGTGAACGGATTTG-3’, Reverse Primer: 5’-TGTAGACCATGTAGTTGAGGTCA-3’. GAPDH (Human) Forward Primer: 5’-TCAACGACCACTTTGTCAAGCTCA-3’, Reverse Primer: 5’-GCTGGTGGTCCAGGGGTCTTACT-3’.

### Western blot analysis

Proteins extracted from the liver tissues and cells were resuspended in RIPA lysis buffer (Applygen Technologies Inc., Beijing, China) mixed with a protease inhibitor cocktail (Roche Diagnostics). Equal amounts of proteins were resolved with SDS-polyacrylamide gels in a mini-gel apparatus (Mini-PROTEAN II, Bio-Rad), and proteins were transferred to a polyvinylidene difluoride membrane (PVDF, Roche Diagnostics, Indianapolis, IN, USA) and incubated overnight with primary antibodies at 4°C. After 3 washes, the membrane was incubated with horseradish peroxidase (HRP)-coupled secondary antibodies for 1 h at room temperature. The membrane was washed again, and the proteins were visualized with an enhanced chemiluminescence (ECL) kit (Lianke, Hangzhou, China). Expression of GAPDH in liver homogenates or cells was routinely evaluated as a loading control. Scanned images were quantified using Quantity One software (Bio-Rad), which created densitometric and volumetric data of the blots. The Band Analysis tools were used to select and determine the background-subtracted density of the bands in all the gels and blots. Cross-section, density distribution, and 3D plotting analyses were used to ensure that the selected single bands were not actually composed of two or more separate bands. Local and global background densities were subtracted from the total band intensity values. Values for each individual sample were calculated by dividing the average sample density by the average density for GAPDH. Data were represented as mean ± SD of three independent experiments.

### Statistical analysis

The SPSS22.0 software was used for the statistical analyses. The experimental data were expressed as the mean ± SD and assessed by two-tailed Student’s T test. Statistical significance indicated by *P*<0.05 and was denoted with asterisks: *P < 0.05; **P < 0.01.

## Results

### Hepatic expression of CAV1 was downregulated in FFA-treated hepatocytes and livers from HFD-fed mice

**Fig 1:**
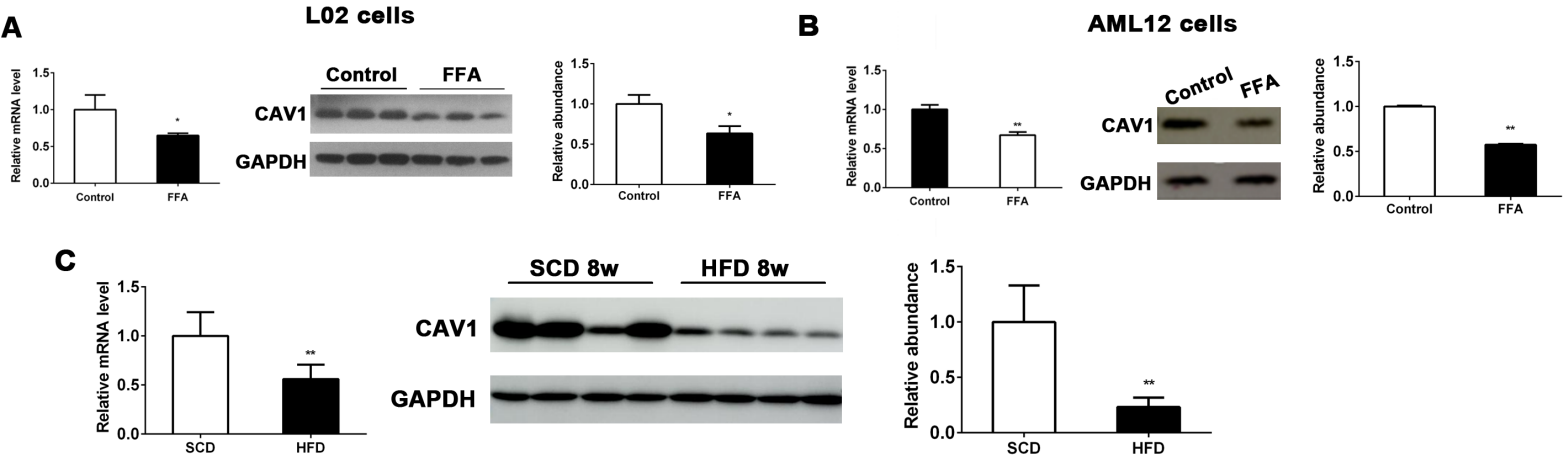
CAV1 expression is downregulated in FFA-treated hepatocytes and in the livers of HFD-fed mice. CAV1 mRNA and protein expression levels were analyzed by qPCR and western blot in L02 cells (A) and AML12 cells (B) treated with or without FFA for 48 h. The results were normalized to the expression level of GAPDH. The results are expressed as the mean ± SD of 3 independent experiments. *P <0.05 and **P <0.01 compared with cells cultured without FFA. (C) Hepatic mRNA and protein expression levels of CAV1 were markedly reduced in mice fed an HFD for 8 weeks. The results are expressed as the mean ± SD of 5 mice per group. *P <0.05 and **P <0.01 compared with mice fed an SCD.

To investigate the potential correlation of CAV1 with metabolic homeostasis in the liver, we first examined CAV1 expression in NAFLD cell models. We observed that both mRNA and protein levels of CAV1 were significantly decreased in FFA-treated human and murine hepatocyte cells (L02 and AML12 cells, respectively) compared with controls (Fig 1A and 1B). To confirm this change, we determined the effects of an HFD on CAV1 expression in the liver. We detected the markedly decreased mRNA and protein expression of CAV1 in the livers of mice fed an HFD for 8 weeks compared to those of SCD-fed mice (Fig 1C). Hepatic CAV1 mRNA and protein expression levels were also decreased in livers of mice fed with HFD for 12 weeks (S1A Fig). Collectively, these results suggested that CAV1 might play a role in the development of diet-induced fatty liver disease.

### CAV1 depletion aggravates fat accumulation in L02 cells and AML12 cells induced by FFA

**Fig 2:**
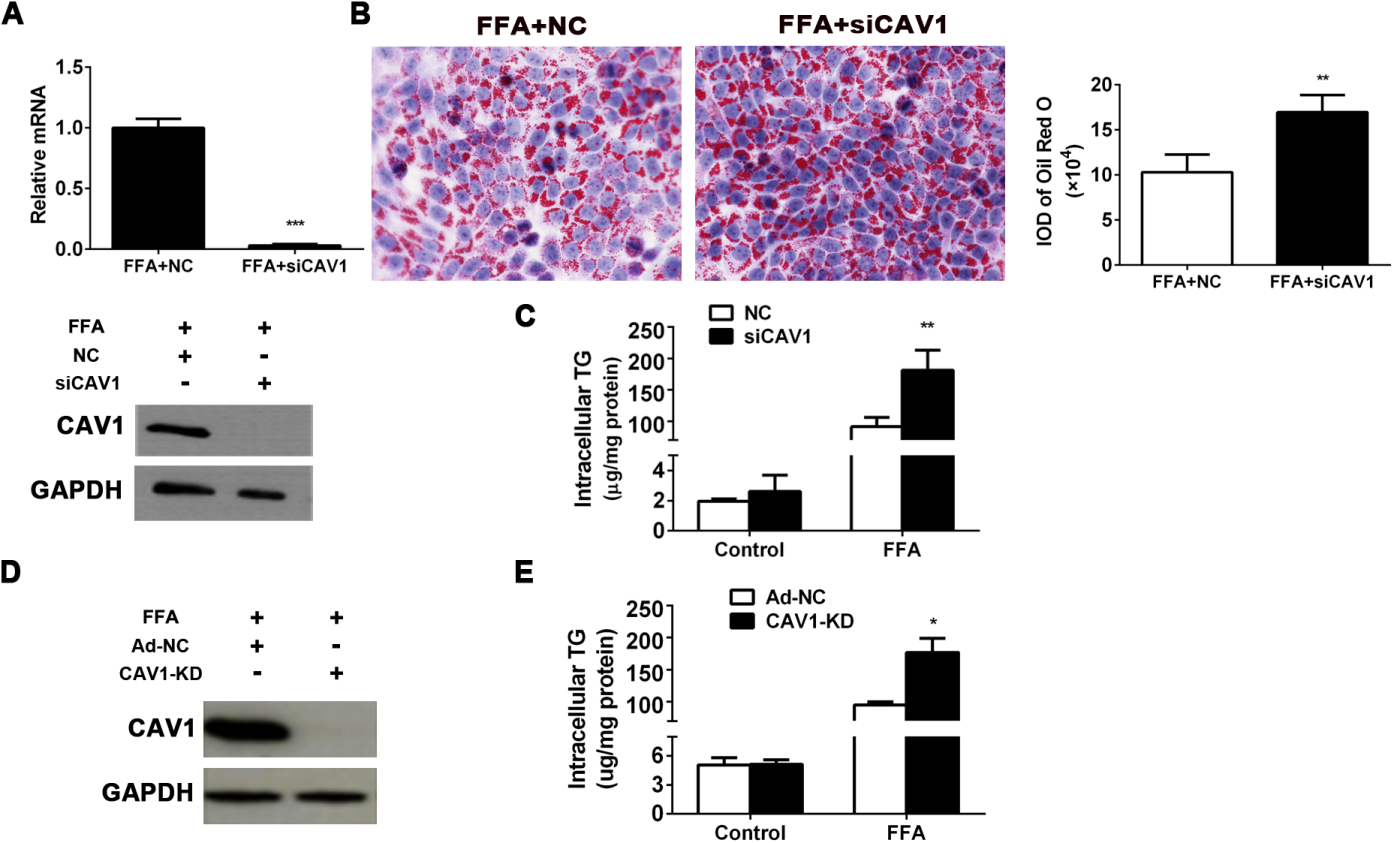
CAV1 depletion aggravates fat accumulation in L02 cells and AML12 cells induced by FFA. CAV1 expression was inhibited using siRNA in L02 cells and stably depleted by applying the CRISPR-Cas9 system by adenovirus recombination in AML12 cells. (A-C) L02 hepatocytes were transfected with scrambled siRNA (NC) or siRNA targeting CAV1 (siCAV1). After transfection for 24 h, cells were challenged with 1 mM FFA for another 48 h. (A) CAV1 mRNA and protein levels in FFA-treated L02 cells transfected with NC or siCAV1. (B) Representative Oil Red O staining of FFA-induced L02 cells with or without CAV1 siRNA transfection. (Original magnification × 400) The average integrated optical density (IOD) of lipid droplets stained with Oil Red O from FFA treated cells was measured with an Image-Pro Plus software. (C) Triglycerides were quantified in L02 cells treated with or without FFA transfected with NC or siCAV1. (D-E) AML12 hepatocytes were transfected with the adenovirus-CRISPR-Cas9 system to stably knockdown CAV1 (CAV1-KD); Ad-NC served as a control. Cells were challenged with 1 mM FFA for 48 h. (D) CAV1 protein levels in FFA-treated AML12 hepatocytes transfected with Ad-NC and CAV1-KD. (E) Triglycerides were quantified from AML12 cells treated with or without FFA transfected with Ad-NC and CAV1-KD. The results are expressed as the mean ± SD of 3 independent experiments. (*P < 0.05, **P < 0.01, and ***P < 0.001).

To examine the effect of CAV1 deficiency at the cellular level, endogenous CAV1 expression was effectively deleted using specific CAV1 siRNA in FFA-treated L02 cells (Fig 2A). Our results indicated that depletion of CAV1 significantly aggravated FFA-induced steatosis in L02 cells as determined by Oil Red O staining (Fig 2B). Besides, fluorescent staining of neutral lipids with BODIPY493/503 showed that the lipid spot area per cell was significantly increased in siCAV1 group L02 cells incubation with FFA (S2A Fig). Intracellular TG measurement revealed a marked elevation caused by CAV1 depletion in FFA-treated L02 cells, while there was no significant difference in TG levels between L02 cell groups treated with control medium (Fig 2C). By combining the CRISPR-Cas9 system with the adenovirus recombination technique, we developed stable CAV1-knockdown (CAV1-KD) AML12 hepatocyte cell lines (Fig 2D) to determine the influence of inherent CAV1 depletion on hepatocyte steatosis. Consistently, intracellular TG contents were significantly increased in CAV1-KD AML12 cells treated by FFA, but CAV1-knockdown doesn’t induce the increased TG levels in AML12 cells treated without FFA (Fig 2E). These results indicate that CAV1 deficiency may aggravate FFA-induced fat accumulation in hepatocytes.

### CAV1 overexpression ameliorated fat accumulation in L02 cells and AML12 cells induced by FFA

**Fig 3:**
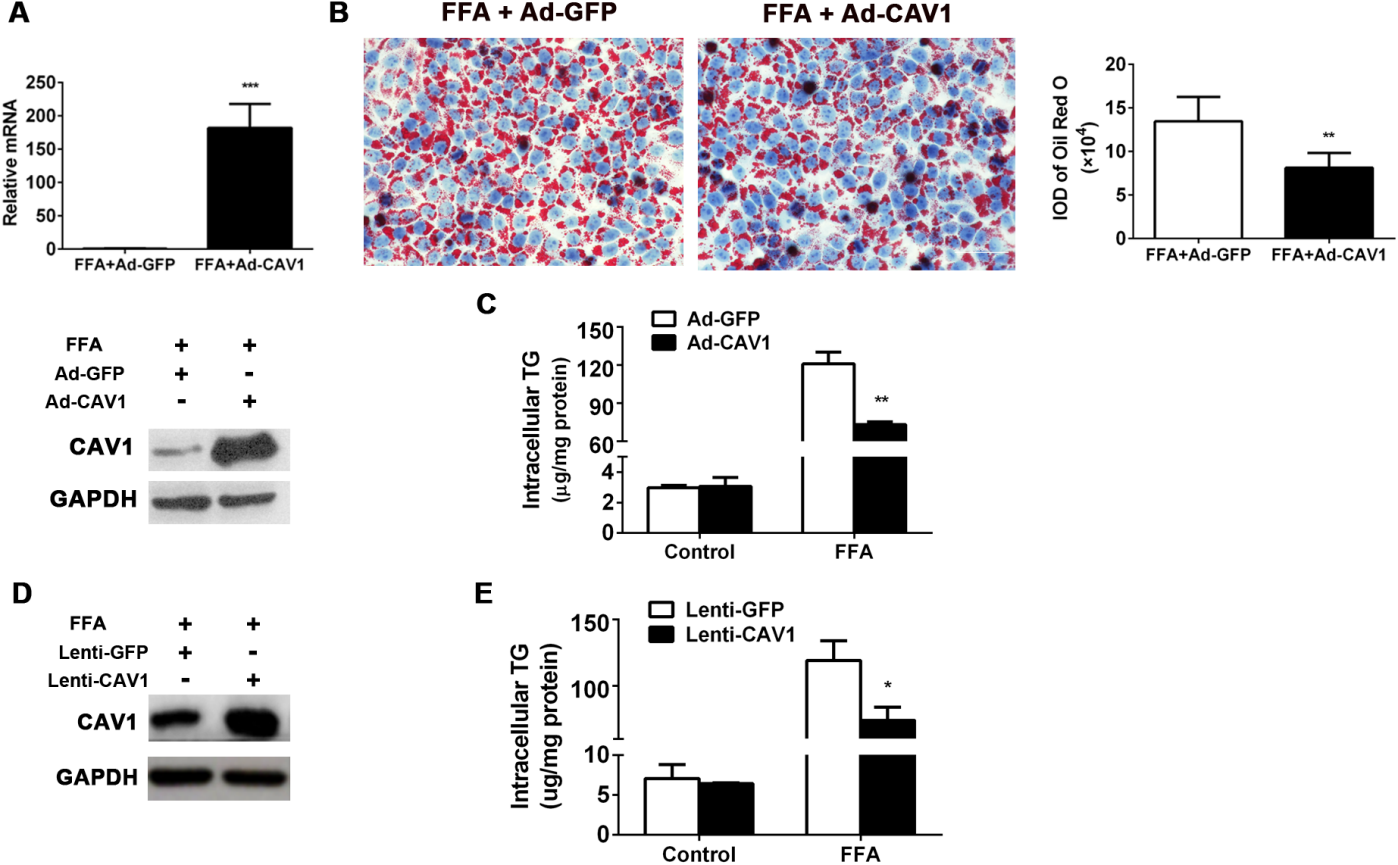
CAV1 overexpression ameliorated fat accumulation in L02 cells and AML12 cells induced by FFA. CAV1 was overexpressed using the adenovirus recombination system via a transient transfection in L02 cells and the lentivirus-constructed stable transfection system in AML12 cells. (A-C) L02 cells were transfected with an adenovirus recombination plasmid containing full-length human CAV1 DNA to overexpress CAV1 (Ad-CAV1); an adenovirus empty vector expressing GFP (Ad-GFP) served as a control. After transfection for 24 h, cells were challenged with 1 mM FFA for another 48 h. (A) CAV1 mRNA and protein levels in FFA-treated L02 cells transfected with Ad-GFP or Ad-CAV1. (B) Representative Oil Red O staining of FFA-treated L02 cells transfected with Ad-GFP or Ad-CAV1. The average integrated optical density (IOD) of lipid droplets stained with Oil Red O from FFA treated cells was measured with an Image-Pro Plus software. (C) Triglycerides were quantified from L02 cells treated with or without FFA transfected with Ad-GFP or Ad-CAV1. (D-E) AML12 hepatocytes were transfected with the lentivirus system containing full-length mouse CAV1 DNA to stably overexpress CAV1 (Lenti-CAV1); a lentivirus empty vector expressing GFP (Lenti-GFP) served as a control. Cells were challenged with 1 mM FFA for 48 h. (D) CAV1 protein levels in FFA-treated AML12 cells transfected with Lenti-GFP or Lenti-CAV1. (E) Triglycerides were quantified from AML12 cells treated with or without FFA transfected with Lenti-GFP or Lenti-CAV1. The results are expressed as the mean ± SD of 3 independent experiments. (*P < 0.05 and ***P < 0.001).

The association of CAV1 gene expression loss with increased lipid accumulation suggested its potentially protective role against FFA-induced hepatocyte steatosis. To test this hypothesis, we performed CAV1 overexpression experiments *in vitro*. Adenoviruses expressing CAV1 (Ad-CAV1) were transfected into L02 cells prior to FFA incubation, while adenoviruses expressing GFP (Ad-GFP) were transfected into cells as a negative control. Enhanced CAV1 expression was confirmed by qPCR and western blot (Fig 3A). Following FFA stimulation, CAV1 overexpression led to a significant improvement of lipid accumulation compared to Ad-GFP-transfected L02 cells as shown by Oil Red O staining (Fig 3B). In line with this, CAV1 overexpression significantly decreased intracellular TG contents in FFA-treated L02 cells, while there was no significant difference in TG levels between L02 cell groups treated without FFA (Fig 3C). Similar results were also observed in the lentivirus construct-mediated, CAV1-overexpressed, FFA-treated AML12 cells (Fig 3D and 3E). Collectively, these gain-of-function results confirmed the ability of CAV1 to sufficiently protect against FFA-induced hepatocyte steatosis.

### Genetic ablation of CAV1 exacerbated hepatic steatosis induced by HFD in mice

To further evaluate and determine the role of CAV1 in the pathogenesis and development of hepatic steatosis, we performed experiments using CAV1-KO mice. Western blot confirmed that the CAV1 protein expression was absent in the livers of CAV1-KO mice (S3A Fig). No obvious histological differences were observed in the livers from ten-week-old CAV1-KO mice and their wild-type (WT) control mice fed a standard chow diet (SCD) (S3B Fig). Body weights and the liver weight/body weight ratio of CAV1-KO mice did not differ from their WT control mice fed on SCD (S3C Fig). The plasma data showed that levels of TC were significantly higher in CAV1-KO mice than in WT mice, whereas TG levels were significantly lower in CAV1-KO mice (S3D Fig). Besides, CAV1-KO mice displayed significantly higher plasma ALT and AST levels (S3E Fig). Consistent with the similar histological appearances between genotypes, no significant differences in the levels of intrahepatic TG and TC were detected in CAV1-KO mice and WT control mice fed on SCD (S3F and S3G Fig).

**Fig 4:**
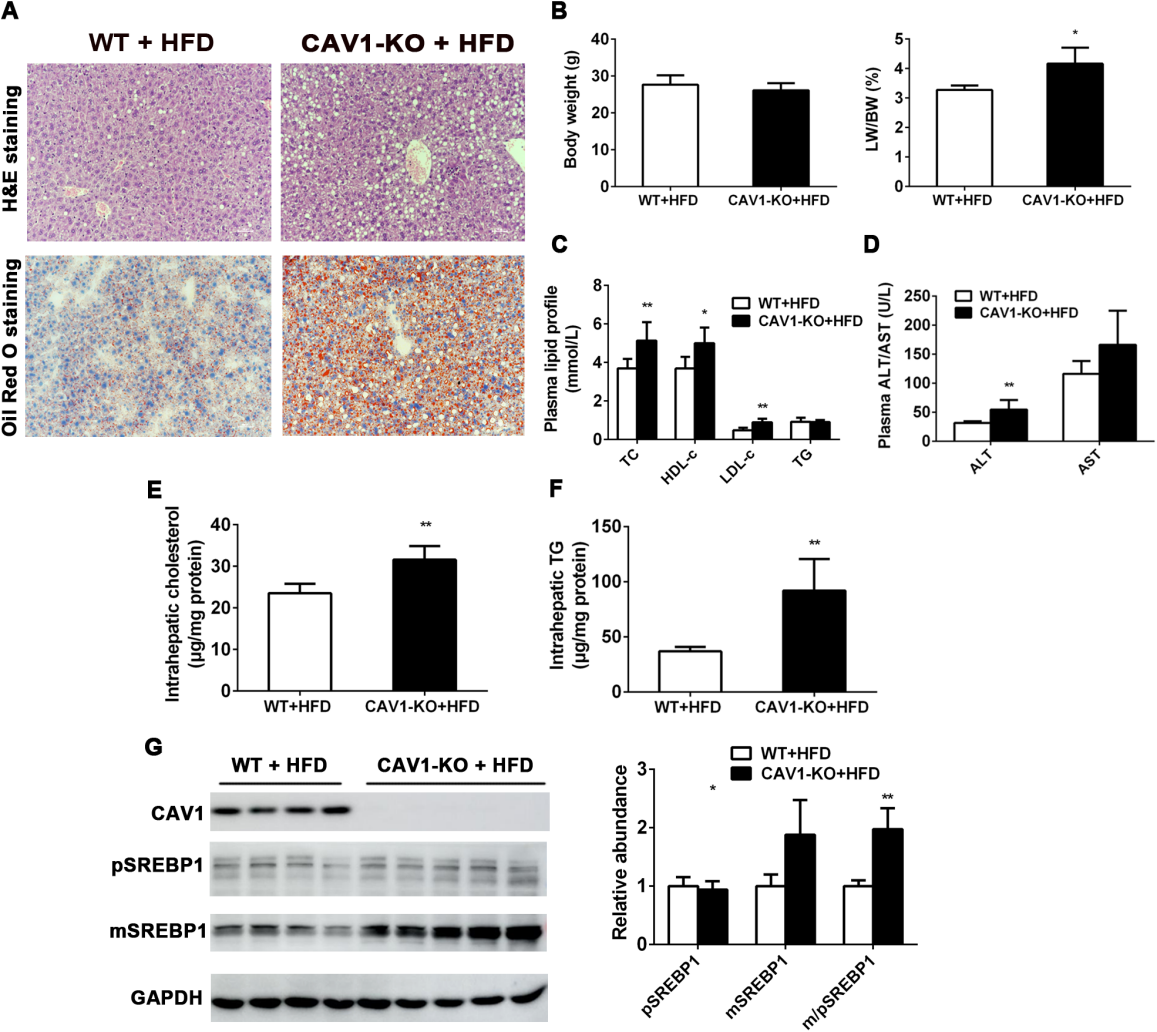
Genetic ablation of CAV1 aggravates HFD-induced hepatic steatosis in mice. CAV1-KO mice and littermate WT mice were fed with an HFD for 4 weeks. (A) Representative hematoxylin and eosin (H&E) staining and Oil Red O staining of sections from the livers of WT and CAV1-KO mice fed an HFD. (Original magnification × 400) (B) Comparison of the body weight (left) and the liver/body ratio (right). (C) The plasma levels of total cholesterol (TC), HDL-c, LDL-c and TG in HFD-fed WT and CAV1-KO mice. (D) The plasma levels of ALT and AST in CAV1-KO mice and littermate WT mice were determined. (E) TC and (F) TG contents were quantified from whole livers of HFD-fed WT and CAV1-KO mice. (G) The protein expression levels of CAV1 and SREBP1 were determined by western blot and quantified with GAPDH as a loading control. pSREBP1 and mSREBP1 denote the precursor and mature forms of SREBP1, respectively. Data are presented as the mean ± SD. The CAV1-KO HFD-fed group (*n* = 5) versus the WT HFD-fed group (*n* = 6); *P < 0.05 and **P < 0.01.

Next we fed WT and CAV1-KO mice with an HFD to assess the effect of HFD exposure on CAV1-regulated hepatic steatosis. Notably, H&E staining and Oil Red O staining revealed severe steatosis with various sizes of lipid deposition in the livers of CAV1-deficient mice fed an HFD for 4 weeks compared to the livers of WT littermates (Fig 4A). Consistent with these histological observations, the liver weight/body weight ratio increased considerably in HFD-fed CAV1-KO mice even though the CAV1 deficiency did not elicit a significant effect on body weight compared to the WT groups (Fig 4B). In addition, levels of TC, HDL-c and LDL-c in plasma were significantly higher in HFD-fed CAV1-KO mice than in WT mice, whereas plasma TG levels were comparable between the two groups (Fig 4C). Elevations in ALT levels occurred with NAFLD and were secondary to hepatocellular inflammation and injury [16]. As shown in Fig 4D, plasma ALT and AST levels exhibited elevated trends in the HFD-fed CAV1-KO mice; however, only the differences in ALT levels were statistically significant, suggesting a greater degree of hepatocyte injury in HFD-fed CAV1-KO mice. Furthermore, hepatic TC contents were significantly higher in HFD-fed CAV1-KO mice (Fig 4E). More strikingly, hepatic TG levels were elevated more than 2-fold in HFD-fed CAV1-KO mice compared to controls (Fig 4F). Parallel with these changes in fat content in the liver, the protein expression of the mature form of sterol response element binding protein-1 (SREBP1), a transcription factor that activates genes involved in lipogenesis, was significantly higher in HFD-fed CAV1-KO mice compared with WT mice (Fig 4G). Moreover, the protein expression of peroxisome proliferator-activated receptor a (PPARα), which regulates fatty acid oxidation (FAO), trended downwards in HFD-fed CAV1-KO mice (S4A Fig), which was consistent with a previous study demonstrating that CAV1-/- mice exhibited impaired PPARα signaling [7]. Taken together, the above data reveal that CAV1 deficiency promotes HFD-induced hepatic steatosis and subsequent hepatic injury.

### Silencing CAV1 expression upregulates genes involved in fatty acid metabolism in the FFA-induced NAFLD *in vitro* model

**Fig 5:**
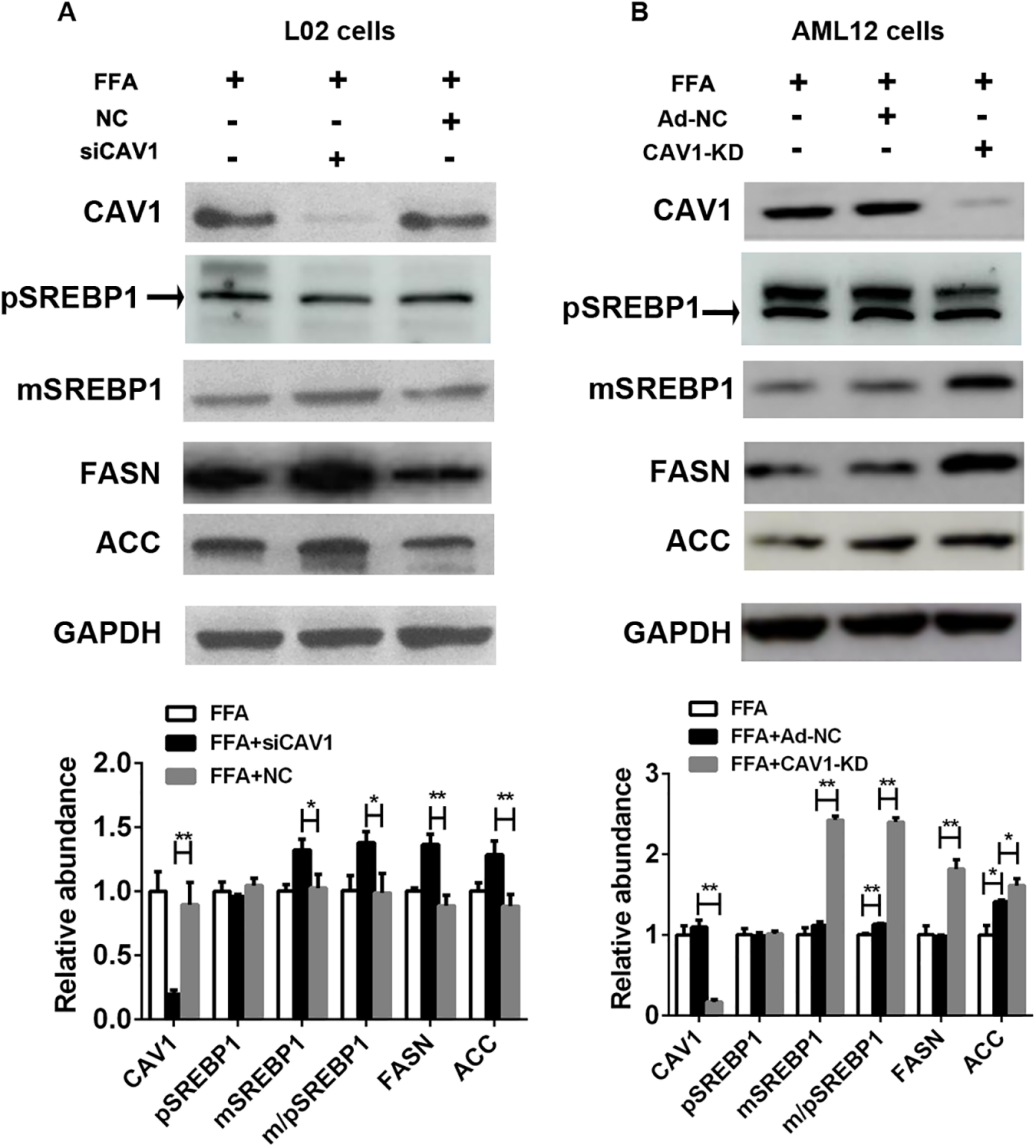
CAV1-knockdown upregulates the protein expression of genes involved in lipid metabolism in the FFA-stimulated L02 cells and AML12 cells. L02 hepatocytes were transfected with scrambled siRNA (NC) or siRNA targeting CAV1 (siCAV1). AML12 hepatocytes were transfected with the adenovirus-CRISPR-Cas9 system to stably knockdown CAV1 (CAV1-KD) while Ad-NC served as a control. Cells were challenged with 1 mM FFA for 48 h. The protein expression levels of fatty acid metabolism genes, including SREBP1, FASN and ACC, were determined by western blot and quantified with GAPDH as a loading control in FFA-treated L02 cells (A) and AML12 cells (B). pSREBP1 and mSREBP1 denote the precursor and mature forms of SREBP1, respectively. The results represent data from 3 independent experiments.

To further address the potential molecular mechanisms underlying the hepatic CAV1 depletion-induced exacerbation of fatty liver disease, we examined the expression of genes regulating fatty acid homeostasis in FFA-treated hepatocytes. Consistent with the findings in CAV-KO mice, protein levels of the mature form of SREBP1 were significantly increased both in siCAV1 L02 cells and CAV1-KD AML12 cells (Fig 5A and 5B). In addition, the protein levels of fatty acid synthase (FASN) and acetyl-CoA carboxylase (ACC) triggered by SREBP1, which drives lipogenesis in the liver, were elevated to a greater extent in siCAV1-transfected L02 cells and CAV1-KD AML12 cells than in controls (Fig 5A and 5B).

### CAV1 overexpression downregulates genes involved in fatty acid metabolism in the FFA-induced NAFLD *in vitro* model

**Fig 6:**
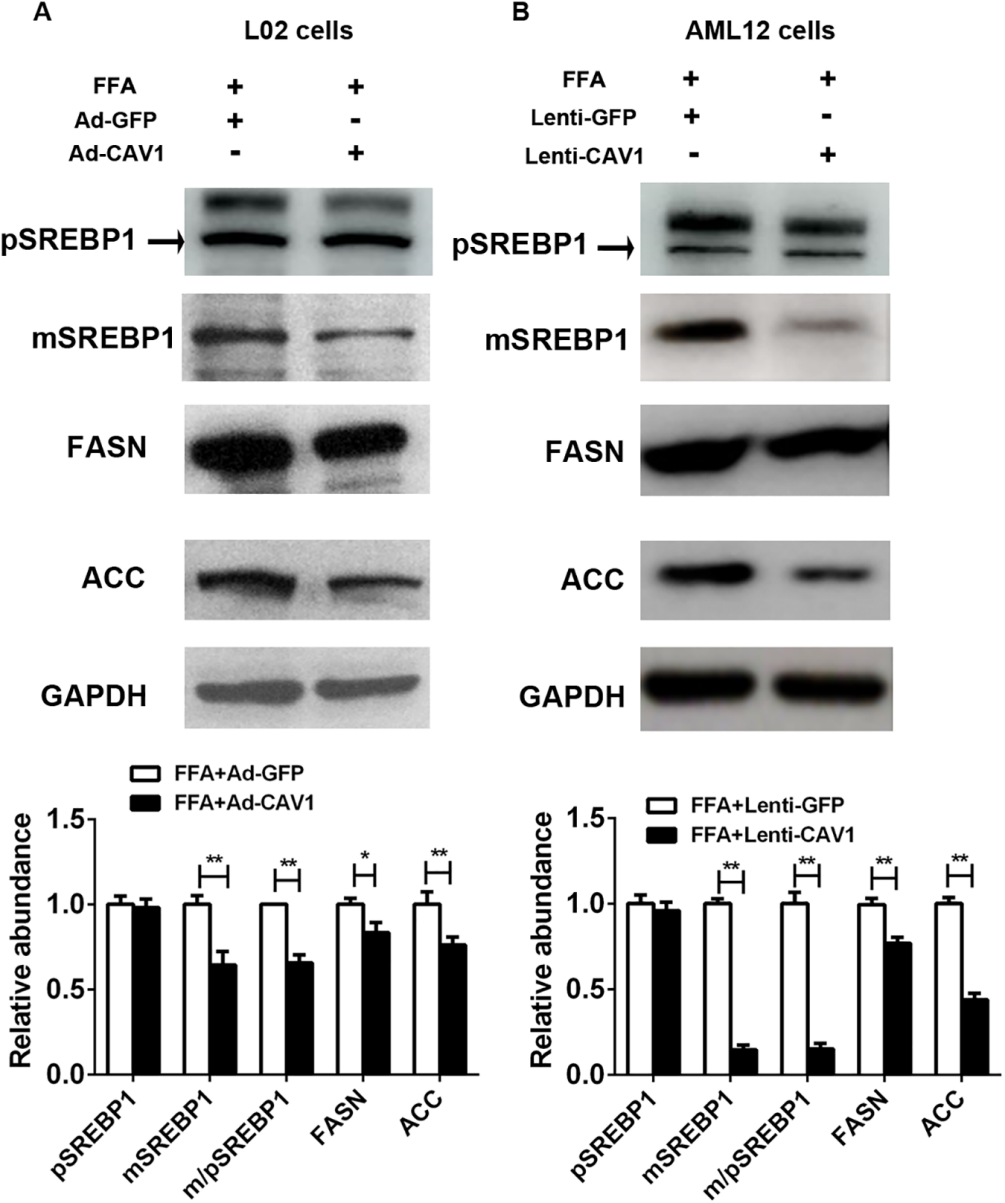
CAV1-overexpression downregulates the protein expression levels of lipid metabolism genes in FFA-stimulated L02 cells and AML12 cells. L02 cells were transfected with adenovirus recombination plasmids containing full-length human CAV1 DNA to overexpress CAV1 (Ad-CAV1), while AML12 hepatocytes were transfected with a lentivirus-constructed system containing mouse full-length CAV1 DNA to stably overexpress CAV1 (Lenti-CAV1). Cells were challenged with 1 mM FFA for 48 h. The protein expression levels of fatty acid metabolism genes, including SREBP1, FASN and ACC, were determined by western blot and quantified with GAPDH as a loading control in L02 cells (A) and AML12 cells (B). pSREBP1 and mSREBP1 denote the precursor and mature forms of SREBP1, respectively. The results represent data from 3 independent experiments.

Next, we checked whether CAV1 overexpression modulated the expression of genes that mediate lipogenesis. Repressed protein expression of the mature form of SREBP1 and its downstream targets, including FASN and ACC, was observed in Ad-CAV1 L02 cells and Lenti-CAV1AML12 cells cultured with FFA (Fig 6A and 6B). These results confirmed that CAV1 improves lipid accumulation in hepatocytes by downregulating lipogenic genes.

## Discussion

As a major structural protein of caveolae, which are involved in lipid homeostasis and endocytosis, CAV1 has emerged as a regulator of liver function, modulating several molecular pathways that lead to the regulation of lipid and glucose metabolism, mitochondrial biology, and hepatocyte proliferation [17].

In this work, we showed that CAV1 expression was downregulated in FFA-induced steatotic L02 and AML12 hepatocytes as well as in the livers of HFD-fed mice. Using *in vitro* CAV1 loss- and gain-of-function studies, the ability of CAV1 to sufficiently protect against FFA-induced hepatocyte steatosis was revealed. Additionally, we showed that mice lacking CAV1 developed more-severe hepatic steatosis when fed an HFD, demonstrating that CAV1 protects against the development of hepatic steatosis and hepatocyte injury that is involved in NAFLD. The mechanism behind this regulation involved impaired SREBP1 signaling and the modulation of PPARα expression.

We first presented new evidence that the CAV1 levels in the liver appear to be critical since its expression was markedly decreased in NAFLD cell and mouse models. Similar results have been observed in a previous study that demonstrated that the downregulated expression of OA/PA induced steatosis in hepatocytes and livers from HFD mice, db/db mice and ob/ob mice, and NAFLD patients [18]. One study reported a major decrease in CAV1 expression in the visceral adipose tissue of obese patients [9]. Since it shares the “common soil” i.e. IR, NAFLD is closely related to T2DM and obesity [19]. Moreover, a potential defect in CAV1 content may be a factor in the development of T2DM in mice [20]. How the expression of CAV1 is regulated remains unclear; Li et al. demonstrated that CAV1 is a direct target of miR199a-5p in hepatocytes. In addition, other mechanisms such as upstream transcription factors (e.g., PKC3 and ETS1) may also partially contribute to the regulation of CAV1 [21,22].

Using several techniques to knockdown and overexpress the CAV1 gene in human and mouse hepatocytes, we observed markedly aggravated and attenuated lipid accumulation, respectively, in the FFA-induced NAFLD *in vitro* models. These current results provide direct evidence for the potentially protective role of CAV1 in hepatic fat accumulation. We thus directly tested whether CAV1 is required for protection against hepatic steatosis *in vivo* by examining mice that lacked CAV1. Systemic loss of caveolin-1 leads to a complex metabolic phenotype, including a substantial decrease in metabolic flexibility [8]. Specifically, our data highlight the significance of the systemic expression of CAV1 for hepatic lipid metabolism. First, we found that the liver weights in relation to the body weights were increased in CAV1-KO mice compared to WT mice fed an HFD, which was consistent with the previous observation that the liver/body weight ratio was elevated in CAV1-null mice under multiple conditions (chow diet, HFD, HFD in addition to alternate-day fasting) [8]. We showed that CAV1-KO mice exhibited more lipid deposition in the liver, possibly originating from abnormal features of hepatic lipid metabolism, such as increased lipogenesis and increased TG and cholesterol synthesis. Genetic ablation of CAV1 has been shown to lead to increased cholesterol content in the liver and mouse embryonic fibroblast mitochondria, which correlates with reduced mitochondrial respiration [23]. This effect might be associated with impaired CAV1 functions, such as the binding and transportation of fatty acids and cholesterol. Without CAV1, free cholesterol accumulates in mitochondrial membranes, increasing membrane condensation and reducing the efficiency of the respiratory chain and intrinsic antioxidant defense and ultimately predisposing CAV1-deficient animals to steatohepatitis [23]. In addition, Asterholm et al. postulated that the metabolic phenotype in the livers of CAV1-/- mice was mainly caused by adipocyte-CAV1 deficiency [8].

However, one study showed that the expression of CAV1 in mice is required for efficient hepatic lipid storage during fasting, liver regeneration, and diet-induced steatosis in three CAV1-/- mouse strains [13]. The reason for this discrepancy remains unknown and may be relevant to diet and environmental factors.

To date, the molecular mechanisms by which CAV1 mediates liver steatosis, especially in diet-induced fatty livers, remain unclear. SREBP1 plays a role in maintaining lipogenic and cholesterologenic enzymes [24]. We observed that the CAV1-deficient livers of HFD-fed mice exhibited greater expression of SREBP1; moreover, enhanced SREBP1 expression in two different and independent CAV1-knockdown hepatocyte steatosis models synergistically supports the notion that the loss of CAV1 accelerates the accumulation of hepatic lipids by inducing SREBP1 transcriptional activity. The upregulation of SREBP1 upon CAV1 knockdown is unexpected, since the aberrant free cholesterol accumulation in endoplasmic reticulum caused by CAV1 genetic deficiency would be predicted to rather curb SREBP1 activation[25]. However, the amount of mature SREBP1 peptide that reaches the nucleus is not primarily controlled by sterol-mediated protease cleavage. SREBP1 promoter can be activated regardless of sterols, instead, transcriptional regulation appears to play the major role and the factors including feed-forward regulation, liver X-activated receptors (LXRs), glucagon, and insulin[26]. We speculate that the regulatory effect of CAV1 on SREBP1 might be in indirect way involving other molecular mediators. Hepatic lipogenesis is mainly regulated by SREBP1, which increases the expression of genes involved in de novo lipogenesis, such as FASN and ACC. Mukherjee et al. found that CAV1 knockdown stimulated lipogenesis and increased the protein level of FASN in the 3T3-L1 and HIB1B adipocyte cell lines [27]. Here, our results showed that both FASN and ACC protein expression was upregulated upon CAV1 knockdown but downregulated upon CAV1 overexpression in FFA-induced hepatocyte steatosis. Taken together, our present data suggest the importance of CAV1-dependent SREBP1 regulation in the development of fatty livers; however, the molecular basis for this regulation remains elusive. Interestingly, CAV1 expression might be regulated by SREBP1. Several studies have revealed that SREBP1 could bind to the sterol regulatory elements (SREs) in the CAV1 gene promoter and subsequently negatively regulate CAV1 expression [28,29]. One recent study supported this conception in which they found SREBP1 knockdown induced by siRNA resulted in a significant increase in CAV1 mRNA level [30]. The study reporting curcumin inhibits ox-LDL-induced cholesterol accumulation in cultured rat vascular smooth muscle cells (VSMC) through increasing the caveolin-1 expression via the inhibition of nuclear translocation of SREBP1 also supported the above findings [31]. The interplay between CAV1 and SREBP1 is still uncertain and future studies are needed to dissect the relationship between CAV1 and SREBP1 signaling in detail.

The second possible molecular mechanism underlying CAV1-regulated hepatic steatosis might lie in the inhibition of PPARα, which is essential in the modulation of lipid metabolism as it activates the mitochondrial and peroxisomal fatty acid β-oxidation pathways [32]. As previously reported, CAV1-/- mice exhibited impaired hepatic PPARα-dependent oxidative fatty acid metabolism [7]. The HFD-fed CAV1-/- mice also showed lower levels of hepatic PPARα expression [8,13].

Another prominent finding in the present work was the elevated transaminases in the plasma of CAV1-KO mice, which reflected a certain degree of liver injury. It has been reported that human cavin-1, which resembles CAV1, is required for caveolae formation[33] and that its deficiency can lead to hepatomegaly and elevated serum transaminases [34]. Thus, it can be speculated that CAV1 plays an important role in protecting against steatosis-induced hepatic impairment.

In conclusion, this study provides a framework for understanding the protective potential of CAV1 against hepatic steatosis and hepatocyte injury in NAFLD, which is probably due to its functions in modulating the expression of lipid metabolism genes. Elucidation of the molecular mechanisms by which CAV1 loss mediates lipid accumulation in liver steatosis may have an important clinical impact, and hence, gene-therapy approaches to overexpress CAV1 might be of utility to ameliorate or even reverse NAFLD in humans.

## Supporting information

S1 Fig CAV1 expression is downregulated in the liver of HFD-fed mice. (A) Hepatic mRNA and protein expression of CAV1 was significantly decreased in mice fed an HFD for 12 weeks. The results are expressed as the mean ± SD of 5 mice per group. **P <0.01 compared with mice fed an SCD.
(TIF)

S2 Fig CAV1 knockdown increases the abundance of lipid droplets induced by FFA in L02 cells. (A) Left panel: Representative BODIPY staining of FFA-induced L02 cells with or without CAV1 siRNA transfection (Original magnification × 200). Lipid droplets were stained with BODIPY 493/503 (green) and nuclei were stained with Hoechst (blue). Right panel: The lipid spot area per cell, expressed as area per pixel (px2), of FFA-induced L02 cells with or without CAV1 siRNA transfection. The data shown were from one representative experiment of six independent repeats. **P < 0.01.
(TIF)

S3 Fig Genetic ablation of CAV1 doesn’t induce hepatic steatosis in SCD-fed mice. (A) The protein expression levels of CAV1 from the livers of ten-week-old CAV1-KO mice and littermate WT mice were determined by western blot. (B) Representative hematoxylin and eosin (H&E) staining of sections from the livers of WT and CAV1-KO mice fed on SCD. (Original magnification × 400) (C) Comparison of the body weight (left) and the liver/body ratio (right). (D) The plasma levels of total cholesterol (TC) and TG in SCD-fed WT and CAV1-KO mice. (E) The plasma levels of ALT and AST in CAV1-KO mice and littermate WT mice were determined. (F) TC and (G) TG contents were quantified from whole livers of SCD-fed WT and CAV1-KO mice. Data are presented as the mean ± SD. The CAV1-KO SCD-fed group (*n* = 5) versus the WT SCD-fed group (*n* = 5); *P < 0.05 and **P < 0.01.
(TIF)

S4 Fig Influence of CAV1-knockout on the expression of fatty acid oxidation genes in the livers of HFD-induced NAFLD mice. (A) The protein expression of PPARα was determined by western blot and quantified with GAPDH as a loading control. Data are presented as the mean ± SD. The CAV1-KO HFD-fed group (*n* = 5) versus the WT HFD-fed group (*n* = 6).
(TIF)
