# Supplementary figures and images for: Caveolin1 protects against diet induced hepatic lipid accumulation in mice
Source: PLoS One. 2017 Jun 1;12(6):e0178748. doi: 10.1371/journal.pone.0178748 (PMC5453590; doi:10.1371/journal.pone.0178748)

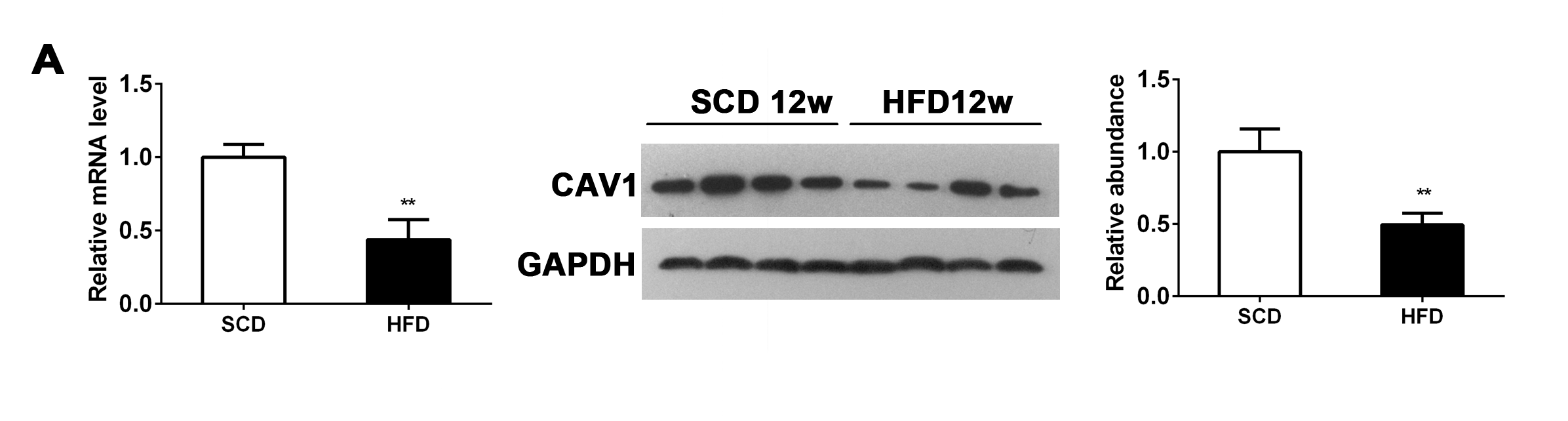

Supplement: S1 Fig — (A) Hepatic mRNA and protein expression of CAV1 was significantly decreased in mice fed an HFD for 12 weeks. The results are expressed as the mean ± SD of 5 mice per group. **P <0.01 compared with mice fed an SCD. (TIF) [file pone.0178748.s001.tif]

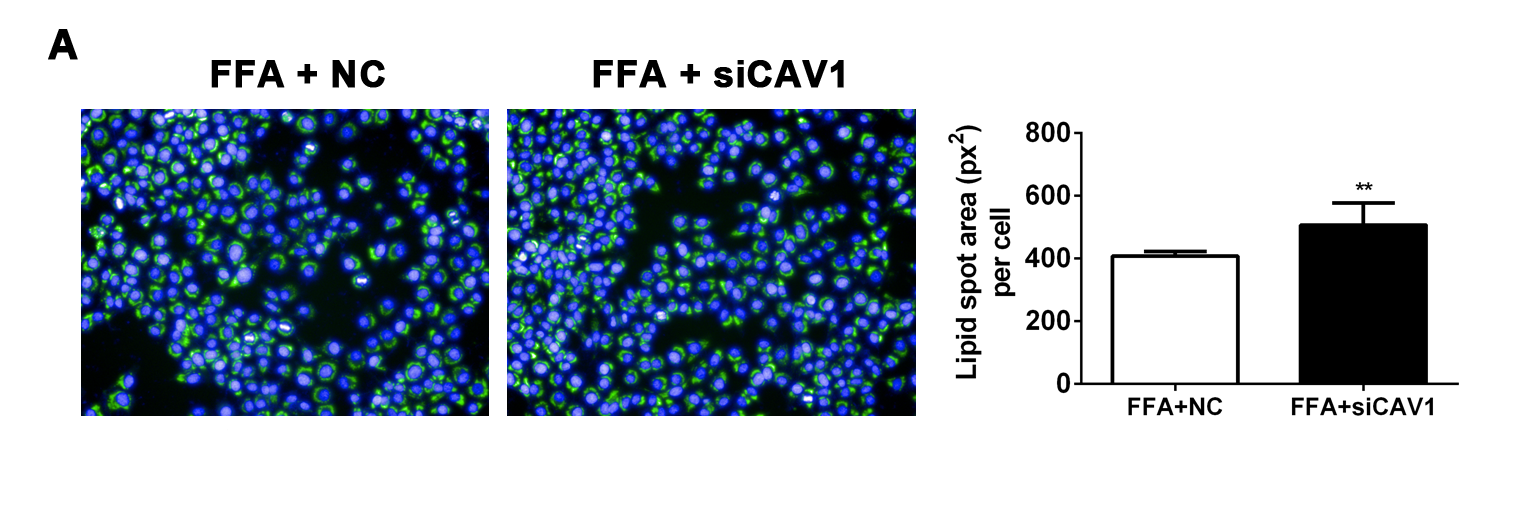

Supplement: S2 Fig — (A) Left panel: Representative BODIPY staining of FFA-induced L02 cells with or without CAV1 siRNA transfection (Original magnification × 200). Lipid droplets were stained with BODIPY 493/503 (green) and nuclei were stained with Hoechst (blue). Right panel: The lipid spot area per cell, expressed as area per pixel (px2), of FFA-induced L02 cells with or without CAV1 siRNA transfection. The data shown were from one representative experiment of six independent repeats. **P < 0.01. (TIF) [file pone.0178748.s002.tif]

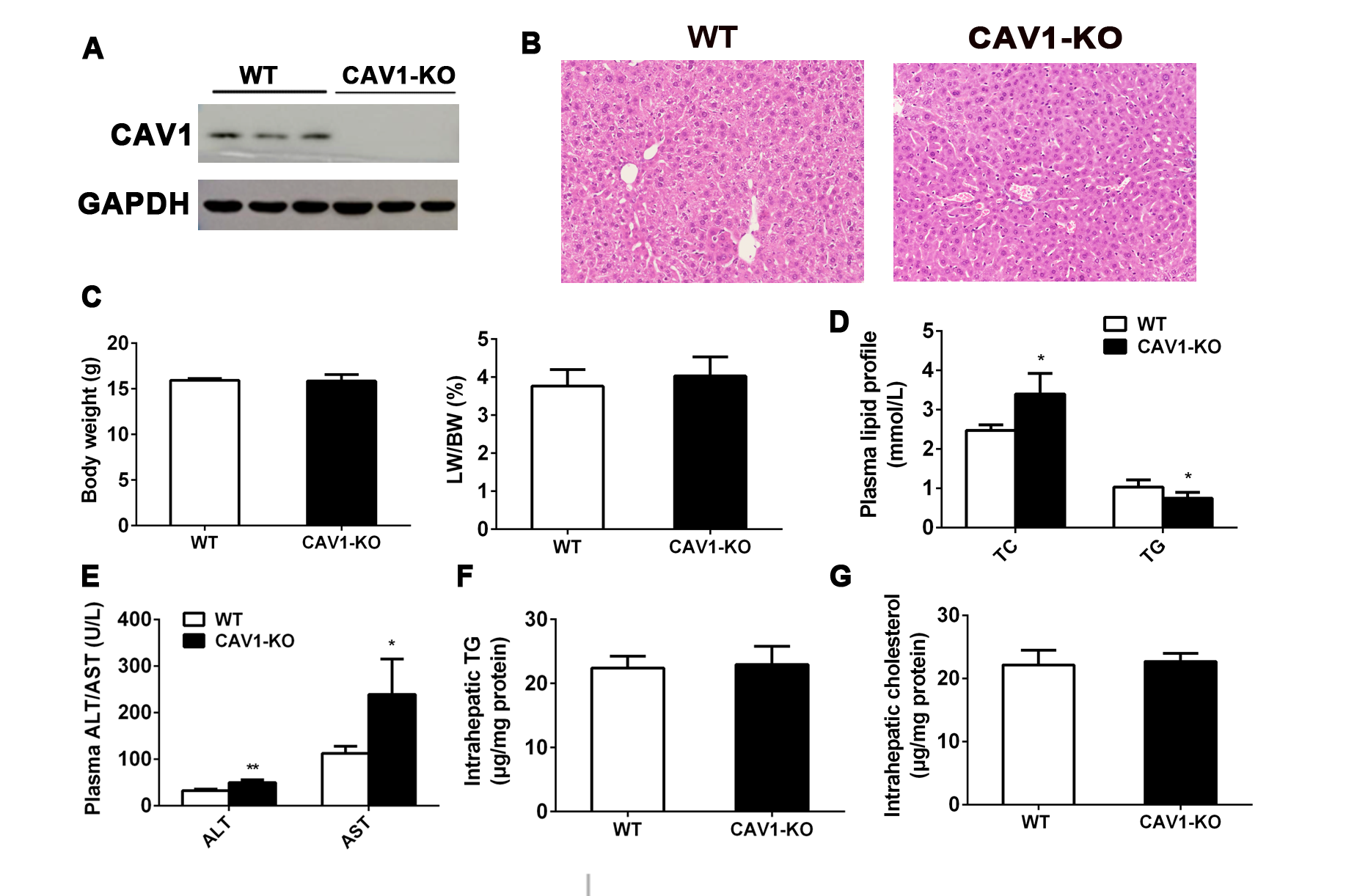

Supplement: S3 Fig — (A) The protein expression levels of CAV1 from the livers of ten-week-old CAV1-KO mice and littermate WT mice were determined by western blot. (B) Representative hematoxylin and eosin (H&E) staining of sections from the livers of WT and CAV1-KO mice fed on SCD. (Original magnification × 400) (C) Comparison of the body weight (left) and the liver/body ratio (right). (D) The plasma levels of total cholesterol (TC) and TG in SCD-fed WT and CAV1-KO mice. (E) The plasma levels of ALT and AST in CAV1-KO mice and littermate WT mice were determined. (F) TC and (G) TG contents were quantified from whole livers of SCD-fed WT and CAV1-KO mice. Data are presented as the mean ± SD. The CAV1-KO SCD-fed group (n = 5) versus the WT SCD-fed group (n = 5); *P < 0.05 and **P < 0.01. (TIF) [file pone.0178748.s003.tif]

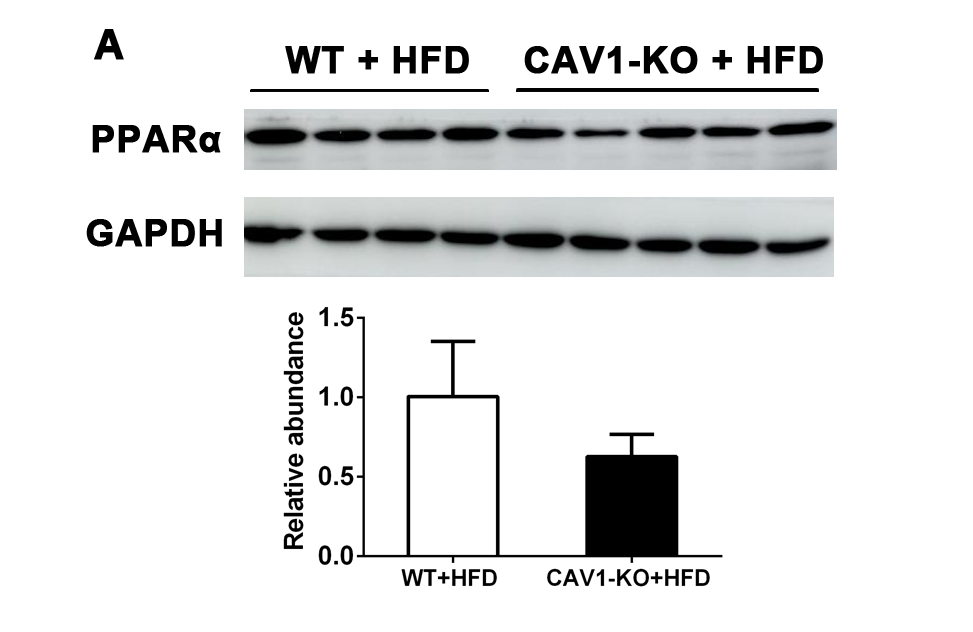

Supplement: S4 Fig — (A) The protein expression of PPARα was determined by western blot and quantified with GAPDH as a loading control. Data are presented as the mean ± SD. The CAV1-KO HFD-fed group (n = 5) versus the WT HFD-fed group (n = 6). (TIF) [file pone.0178748.s004.tif]
